# Impact of COVID-19 on healthcare-associated infections in Canadian acute-care hospitals: Interrupted time series (2018–2021)

**DOI:** 10.1017/ash.2023.390

**Published:** 2023-09-29

**Authors:** Anada Silva, Jessica Bartoszko, Joëlle Caye, Kelly Baekyung Choi, Robyn Mitchell, Linda Pelude, Jeannette Comeau, Susy Hota, Jennie Johnstone, Kevin Katz, Stephanie Smith, Kathryn Suh, Jocelyn Srigley

## Abstract

**Background:** Data regarding the effects of the SARS-COV-2 (COVID-19) pandemic on healthcare-associated infections (HAIs) in Canadian acute-care hospitals are limited. We examined the impact of the COVID-19 pandemic on HAIs and antimicrobial resistant organisms in hospitals participating in the Canadian Nosocomial Infection Surveillance Program. **Methods:** We analyzed 13,406 HAIs including adult mixed intensive care unit (ICU) central-line–associated bloodstream infections (CLABSIs), and healthcare-associated (HA) *Clostridioides difficile* infection (CDI), methicillin-resistant *Staphylococcus aureus* (MRSA) bloodstream infections (BSI), vancomycin-resistant *Enterococcus* (VRE) BSI, and carbapenemase-producing Enterobacterales (CPE) infections collected using standardized case definitions and questionnaires from 29–64 hospitals participating in the Canadian Nosocomial Infection Surveillance Program (CNISP) from January 2018 to December 2021. We used a generalized linear mixed model with quasi-Poisson distribution to assess step and slope changes in monthly HAI rates between the pre–COVID-19 pandemic period (January 1, 2018–February 29, 2020; 26 time points) and the COVID-19 pandemic period (March 1, 2020–December 31, 2021; 22 time points). Results were reported as incidence rate ratios (IRRs) with 95% confidence intervals (CIs) and adjusted for seasonality, hospital clustering, and hospital characteristics of interest. **Results:** In the CNISP network, 7,352 (55%) HAIs were reported in the prepandemic period and 6,054 (45%) in the pandemic period. Median age was significantly younger during the pandemic period compared to the prepandemic period among patients with HA-CDI, HA-MRSA BSI, and adult mixed ICU CLABSIs, and more than half of cases among all reported HAIs were male (range, 52%–65%). The 30-day all-cause in-hospital mortality rate did not significantly change between the prepandemic and pandemic periods for all reported HAIs and was highest among HA-VRE BSIs (34%). Modeling results indicated that the COVID-19 pandemic was associated with an immediate increase in HA-CDI and adult mixed ICU CLABSI rates whereas HA-MRSA BSI, HA-CPE and HA-VRE BSI rates immediately decreased. However, pandemic status did not have a statistically significant lasting impact on monthly rate trends for all reported HAIs after adjusting for seasonality, clustering, and hospital covariates (Fig. 1 and 2). Adjusted IRRs for all HAIs ranged from 1.00 to 1.01 (95% CI, 0.94–0.99 to 1.01–1.05).

**Figure:**
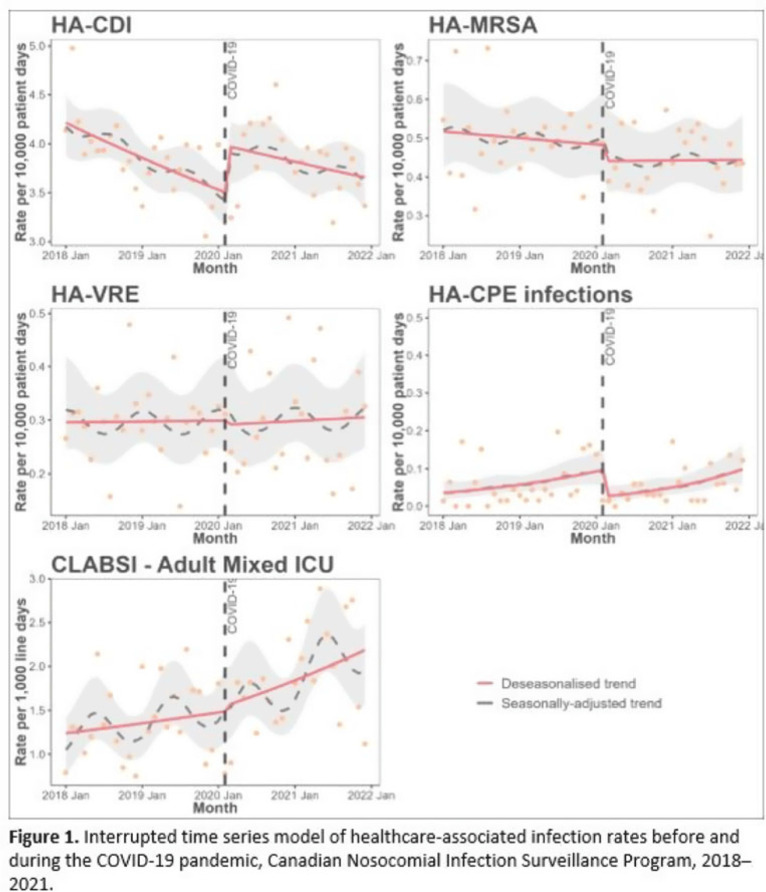

**Figure:**
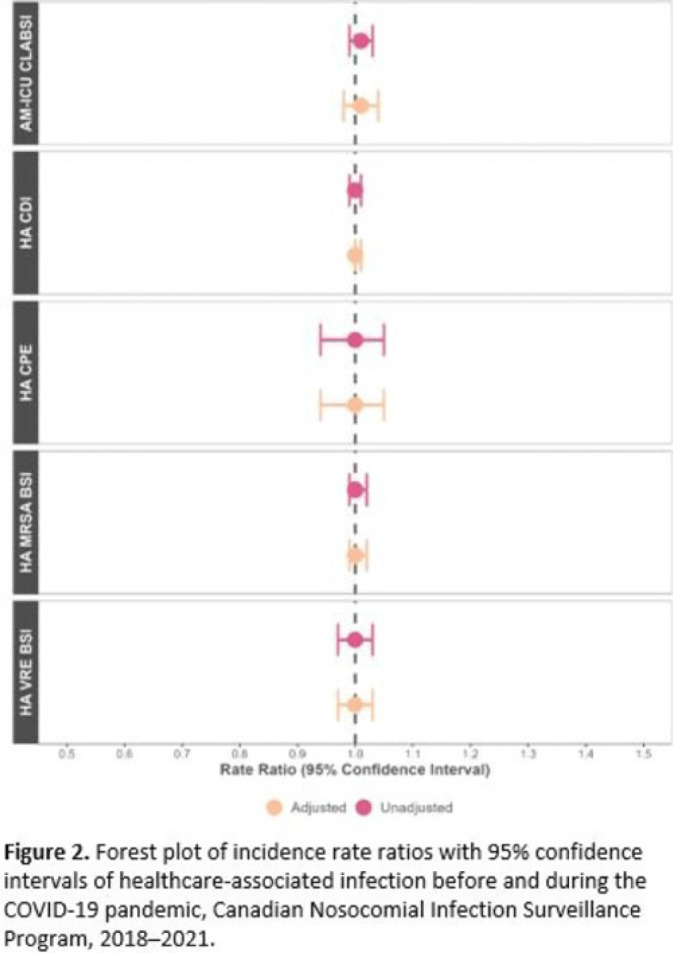

**Conclusions:** Although the COVID-19 pandemic placed a significant burden on the Canadian healthcare system, the immediate impact on monthly rates of HAIs in Canadian acute-care hospitals was not sustained over time. Understanding the epidemiological effects of the COVID-19 pandemic in the context of changing patient populations, and clinical and infection control practices, are essential to inform the continued management and prevention of HAIs in Canadian acute-care settings.

**Disclosures:** None

